# Fructans Are Differentially Distributed in Root Tissues of Asparagus

**DOI:** 10.3390/cells9091943

**Published:** 2020-08-22

**Authors:** Katja Witzel, Andrea Matros

**Affiliations:** 1 Leibniz Institute of Vegetable and Ornamental Crops, Großbeeren, 14979 Brandenburg, Germany; Witzel@igzev.de; 2 ARC Centre of Excellence in Plant Energy Biology, Food and Wine, School of Agriculture, University of Adelaide, Urrbrae, SA 5064, Australia

**Keywords:** asparagus, fructan, fructosyltransferases, HPAEC-PAD, inulin, MALDI-MSI, neoseries-type, proteomics, roots, vegetable

## Abstract

Inulin- and neoseries-type fructans [fructooligosaccharides (FOS) and fructopolysaccharides] accumulate in storage roots of asparagus (*Asparagus officinalis* L.), which continue to grow throughout the lifespan of this perennial plant. However, little is known about the storage of fructans at the spatial level in planta, and the degree of control by the plant is largely uncertain. We have utilized mass spectrometry imaging (MSI) to resolve FOS distribution patterns in asparagus roots (inner, middle, and outer tissues). Fructan and proteome profiling were further applied to validate the differential abundance of various fructan structures and to correlate observed tissue-specific metabolite patterns with the abundance of related fructan biosynthesis enzymes. Our data revealed an increased abundance of FOS with higher degree of polymerization (DP > 5) and of fructopolysaccharides (DP11 to DP17) towards the inner root tissues. Three isoforms of fructan:fructan 6G-fructosyltransferase (6G-FFT), forming 6G-kestose with a β (2–6) linkage using sucrose as receptor and 1-kestose as donor, were similarly detected in all three root tissues. In contrast, one ß-fructofuranosidase, which likely exhibits fructan:fructan 1-fructosyltransferase (1-FFT) activity, showed very high abundance in the inner tissues and lower levels in the outer tissues. We concluded a tight induction of the biosynthesis of fructans with DP > 5, following a gradient from the outer root cortex to the inner vascular tissues, which also correlates with high levels of sucrose metabolism in inner tissues, observed in our study.

## 1. Introduction

Asparagus (*Asparagus officinalis* L.) is a worldwide-consumed perennial vegetable with a high nutritional value and low calorie intake. Consumed spears are rich in polyphenols, flavonoids, ascorbic acid, as well as amino acids [1], while roots are traditionally used as a medicinal product, mainly due to their accumulation of saponins and fructans [2]. Asparagus grows from a root system of fleshy storage roots attached to an underground stem (rhizome). Small feeder roots attached to storage roots absorb nutrients and water and are short-lived, while storage roots continue to grow throughout the lifespan of an asparagus stand (15 years and more). Storage roots accumulate mainly fructans as storage carbohydrates [3,4].

Fructans consist of repeating fructose residues linked to a sucrose unit. They are classified according to the position of the sucrose moiety (internal glucose residue, neoseries-type), the kind of linkage between the fructose residues [β (2,1), inulin; β (2,6), levan or containing both β (2,1) and β (2,6)-d-fructosyl units, graminan-type] and the chain lengths [5]. Fructans can form polymers [with a degree of polymerization (DP) ≥ 10] or oligomers with DP3-9, also referred to as fructooligosaccharides (FOS). Here, the term fructans is used when no differentiation was made between FOS or fructan polymers. 

Fructan biosynthesis genes are of polyphyletic origin and evolved at least four times during plant diversification: (i) in the Asterales (inulin-type fructans), e.g., chicory; (ii) in the Poales with further distinction between cool-season grasses (mainly levan and neoseries-type fructans, e.g., ryegrass) and cereals (predominantly graminan-type fructans, e.g., wheat and barley); (iii) in the Asparagales that mainly accumulate inulin and neoseries-type fructans; and (iiii) in the Buxales (both levan- and graminan-type fructans), e.g., Pachysandra species [6]. Several structures of inulin and neoseries-type fructans have been elucidated in asparagus roots earlier [4,7], and related biosynthesis and hydrolyzing enzymes were identified [8,9,10].

More recently, attention has been paid to the tissue-specific abundance and the various roles of fructans in plants according to their degree of branching and DP level. A causal relation of tolerance against abiotic stress, such as water limitation [11,12], cold [13] and salt stress [14], with an increased tissue-specific accumulation of fructans with higher DP, was reported for several plants species, e.g., perennial ryegrass, *Aloe barbadensis* Miller, and transgenic tobacco. Thereby, the protective effects have been supposed to be based on membrane stabilization and reduction of oxidative stress by those fructan types [15,16]. Similarly, a peak in accumulation of short inulin-type fructans in transfer tissues of developing barley grain during the storage phase has been discussed as probably being related to the protection of the transfer membranes [17]. Increased fructan accumulation was also reported during flowering and seed development, e.g., in *Campanula rapunculoides* [18] and onions [19]. Based on the observation that petals in daylily [20] and *C. rapunculoides* [18] as well as leaves of *Phippsia algida* [21] rapidly degrade fructans upon flower opening, a role for fructans in the expansion of flowers was suggested. Also, the high accumulation of inulin-type fructans in different parts of rhizophores from *Chrysolaena obovata* was reported, while the biosynthetic activity was highest in the distal region (presenting the younger tissues) during the vegetative phase, and the fructan mobilization activity was highest in the proximal region (presenting buds for the development of new shoots) at the sprouting phase [22]. These results have been recently shown to correlate with endogenous phytohormone concentrations (abscisic acid and indoleacetic acid) in proximal and distal segments of *C. obovata* rhizophores [23]. At the level of tissue resolution, inulin-type fructans in *Taraxacum officinale* [24] and *Campuloclinium chlorolepis* [25], as well as levan-type fructans in *Gomphrena marginata* Seub. [26], were shown to preferentially accumulate in vessel elements (phloem and xylem) of roots, while differences in the DP level were not investigated in these studies. In contrast, fructans in asparagus roots are still discussed without differentiation of the structure or tissue-specific abundance, which we aim to investigate in this study.

The restrictive occurrence of proteins and metabolites is challenging for analytical chemistry, and information about the localization of proteins and compounds is lost when homogenized sample material is investigated. Matrix-assisted laser desorption ionization mass spectrometric imaging (MALDI MSI) has been widely applied to track the temporal and spatial dynamics of metabolite accumulation in situ without the need for labelling or derivatization. The versatility of MSI has been well demonstrated for plant-derived metabolites, such as carbohydrates, lipids, glucosinolates and phenylpropanoids [27], and specific applications for oligosaccharides such as β-glucan and arabinoxylan [28] as well as fructans [17,29] were reported, providing novel insights into cell type-specific metabolite compartmentation. With respect to proteome studies, an increasing number of cell type-specific approaches have been performed to understand the plant’s response to certain stresses and plant development, taking into account the disparate cell type-specific dynamics [30,31,32]. Therefore, the objective of our present study was to gain insight into the compartmentalization of biosynthetic pathways related to fructan metabolism to enhance our understanding of the intricate regulation of fructan accumulation in asparagus storage roots at the tissue level.

**Scheme 1:**
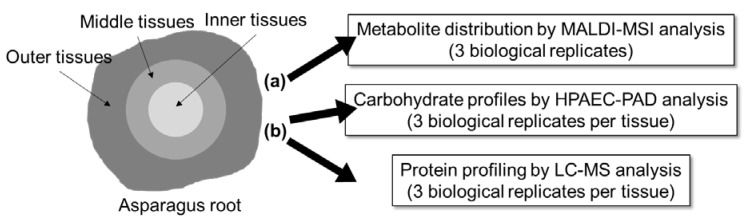
Experimental setup. (**a**) Cross-sections were prepared from asparagus roots and analyzed by MALDI-MSI. (**b**) From such cross-sections, outer, middle, and inner root tissues were punched out and utilized for carbohydrate profiling and protein profiling analysis.

We sought to characterize the spatial localization of fructans in asparagus storage roots by the complementary application of MALDI MSI, fructan profiling, and proteome profiling. In our approach, roots were dissected into three tissues: (i) the inner tissue sample consisting of the vasculature and inner cortex, (ii) the middle tissue sample derived from the cortex, and (iii) the outer tissue sample consisting of the multiseriate epidermis, a uniseriate suberized exodermis and outer cortex (Scheme 1). We show that the accumulation of inulin- and inulin-neoseries-type FOS with higher DP and fructopolysaccharides varies between the different root regions, showing a gradient for increased abundance in inner root tissues, which corresponds with the pattern observed for related fructan biosynthesis enzymes. The potential role of the fructan gradient is discussed regarding the functions of the different root tissues.

## 2. Materials and Methods

### 2.1. Plant Material and Tissue Preparation

Cultivation of asparagus (*Asparagus officinalis* L. ‘Backlim’) was performed as described earlier in detail [33]. Seeds were germinated at 25 °C in the dark until the first stem emerged. Seedlings were then exposed to 25/20 °C; 75/85% relative humidity and a 12/12 h day-night-cycle with a light intensity of ca. 400 µMol m^−2^ s^−1^ and watered as required. After development of the third stem, plants were transferred to pots and exposed to 23/18 °C and 75/85% relative humidity in day-night-cycle with a 16/8 h photoperiod. Three independent plant cultivations were performed.

Two months after sowing, at the fern growth phase, plants were harvested. The substrate was carefully removed from the roots and 1 cm cuttings of storage roots, taken from the middle to upper part of the root system, were snap frozen in liquid nitrogen and stored at −80 °C for further analysis.

Root cross-sections were analyzed using MALDI-MSI (see further below). Carbohydrate and protein profiling were done on core cuttings of root cross-sections (Scheme 1, Figure S1). For this, samples were transferred to a cryostat (CM3050S, Leica, Wetzlar, Germany) with the chamber cooled to −20 °C and the sample holder to −18 °C. The root pieces were fixed to a sample plate using optimal cutting temperature medium (Leica).

Cross-sections were cut from the roots with a razor blade at a thickness of 150 µm. Sequentially, core pieces were punched out of the frozen cross-sections using two coring tools with 1.2 mm and 3 mm diameter (Harris Uni-Core, Sigma Aldrich, Taufkirchen, Germany, Figure S1). Cuttings from roots of three plants, from each of the three independent plant cultivations, were pooled to give one biological replicate.

### 2.2. Mass Spectrometry Imaging

Cross-sections were cut from intact root pieces with a razor blade at a thickness of 16 µm. These sections were immediately thaw mounted onto a conducting indium tin oxide-coated glass slide (ITO slide, Bruker Daltonik, Bremen, Germany) and transferred into a desiccator for 15 min until dryness. Teaching marks were placed in the corners of the glass slide using correction fluid (Tipp-Ex GmbH & Co. KG, Eschborn, Germany) and the image of the slide was captured by a Perfection V700 Photo scanner (Seiko Epson Corporation, Shinjuku, Tokyo, Japan) at 4800 dpi for MSI measurements at a resolution of 40 µm. The matrix was 2, 5-dihydroxybenzoic acid (DHB, Sigma Aldrich) diluted to 30 g L^−1^ in 50% methanol (0.2% trifluoroacetic acid, Sigma Aldrich) and was applied to the slide surface by an ImagePrep device (Bruker Daltonik) using the pre-set method for this matrix.

MSI experiments were performed using an ultrafleXtreme MALDI-time of flight (TOF) instrument (Bruker Daltonik) run in positive ionization mode. The laser raster was set to 40 µm and the *m/z* range was set to 200–2000, with matrix suppression up to *m/z* 180. Sample rate was 0.63 Gs s^−1^ and 1000 shots per raster spot were acquired. For measurements performed in positive mode, the method was calibrated with a mix of Peptide Calibration Standard II (Bruker Daltonik) and a polyethylene glycol mixture (1:1 mixture of PEG 200 and 600, diluted 1:300 in 30% *v/v* acetonitrile and 0.1% *w/v* trifluoroacetic acid). The laser power was adjusted according to the requirements of each experiment. Measurements were performed by flexControl v3.4 software (Bruker Daltonik) and flexImaging v4.0 software (Bruker Daltonik). From each of the three independent plant cultivations, roots of two plants were analyzed with at least two tissue sections per plant.

Tandem mass spectrometry measurements (Lift mode, ultrafleXtreme MALDI-TOF instrument) of selected molecular ions were performed directly on the root sections and annotation of oligosaccharides was based on obtained fragment ion pattern (Figures S2 and S3).

### 2.3. Oligosaccharide Extraction and Profiling

Root tissue preparation is described in Section 2.1. Soluble sugars were extracted in 500 µL of 80% ethanol at 85 °C for 30 min followed by 500 µL of Milli-Q water at 85 °C for 30 min on a mixer (700 rpm) and supernatants combined; method adopted from [34]. Extracts were then dried completely and diluted with water to a final ratio of 1:64,000 (*w*/*v*, mg/µL). Twenty-five microliters per sample were analyzed by high pH anion exchange chromatography with pulsed amperometric detection (HPAEC–PAD) on a Dionex ICS-5000 system (Thermo Scientific, Bremen, Germany) using a DionexCarboPAC^™^ PA-20 column (3 × 150 mm) with a guard column (3 × 50 mm) kept at 30 °C and operated at a flow rate of 0.5 mL min^−1^. The eluents used were (A) 0.1 M sodium hydroxide and (B) 0.1 M sodium hydroxide with 1 M sodium acetate. The gradient used was: 0% (B) from 0–2 min, 30% (B) from 2–55 min, 100% (B) from 55–56.5 min, and 0% (B) from 57.5–58.5 min. Detector temperature was maintained at 20 °C, data collection was at 2 Hz and the Gold Standard PAD waveform (std. quad. potential) was used. 

Data acquisition, processing, and peak integration were performed using the Chromeleon™ version 7.1.3.2425 software (Thermo Scientific). Compounds were annotated based on available analytical standards. Glucose, fructose, sucrose, raffinose, 1-kestose, maltose, maltodextrin, nystose, and mixtures of inulin from chicory (DP 2-60) and levan from *Erwinia herbicola* were purchased from Sigma Aldrich, while 1,1,1-kestopentaose was obtained from Bio-Strategy (Adelaide, South Australia, Australia). Further, inulin and neoseries-type fructans were isolated from onions and barley grain [35]. In addition, fructan-related chromatographic peaks were identified based on fructanase digestion and mild acid hydrolysis [35] as well as based on available literature [9,36,37,38]. A total of 57 peaks have been annotated (Table 1).

### 2.4. Proteome Profiling

Preparation of root tissues is described in Section 2.1. Proteins present in the three root regions were extracted using RapiGest SF (Waters, Eschborn, Germany), reduced and digested following the method of Kaspar et al. [39]. Desalting of peptides was done using Pierce™ Peptide Desalting Spin Columns (, Thermo Scientific) following the manufacturer’s instructions. Peptides were re-suspended in 2% acetonitrile/0.1% trifluoroacetic acid to a concentration of 100 ng µL^−1^ and 6 µL of protein digest were analyzed using nanoflow liquid chromatography on a Dionex UltiMate 3000 system (Thermo Scientific) coupled to a Q Exactive Plus mass spectrometer (Thermo Scientific) as described previously [40], with the following modifications. Peptides were separated using a Acclaim PepMap 100 C18 analytical column (75 µm × 25 cm, 2 µm particle size, 100 Å pore size) and eluted via a 100 min gradient from 2% to 44% solvent B (80% acetonitrile). Each sample was measured in triplicate (MS scan range 375–1500). The raw files were processed using Proteome Discoverer 2.4 and Sequest HT search engine (Thermo Scientific), searching the NCBI *A. officinalis* Annotation Release 100 (as released on 1 March 2017, https://www.ncbi.nlm.nih.gov/datasets/genomes/?txid=4685) and subsequently, the SwissProt plant database (as of April 1st 2020, https://www.uniprot.org/uniprot/?query=reviewed:yes%20taxonomy:33090). Precursor ion mass tolerance was set to 10 ppm and fragment ion mass tolerance was set to 0.02 Da. False discovery rate (FDR) target values for the decoy database search of peptides and proteins were set to 0.01 (strict level for highly confident identifications). Protein abundance quantification was done using the summed abundance method. Missing values were filled by low abundance resampling (missing values are replaced automatically with random values sampled from the lower 5% of all detected values). The result lists were then filtered and only proteins were kept for further investigation that fulfilled the following characteristics: identified by at least two peptides or by one peptide representing at least 10% protein coverage. The 10% level was chosen since it allowed the inclusion of small proteins (10–30 kDa) that might be difficult to cleave by trypsin due to the lack of respective amino acids in the protein sequence. Differential protein expression was validated using t-test (*p* > 0.05, Benjamini-Hochberg corrected for FDR) after an analysis of variance (ANOVA) test, implemented in the Proteome Discoverer software (Thermo Scientific). Functional annotation of proteins was performed using the BlastKOALA tool (https://www.kegg.jp/blastkoala/) and the Kyoto Encyclopedia of Genes and Genomes (KEGG) pathway database [41].

Proteome raw data has been deposited at MassIVE (https://massive.ucsd.edu/ProteoSAFe/static/massive.jsp?redirect=auth) under the dataset ID MSV000085670.

## 3. Results

### 3.1. Mass Spectrometry Imaging Revealed Differential Tissue Abundance Pattern for Fructans

Asparagus storage roots were sampled at the fern growth phase. The matrix-assisted laser desorption/ionization (MALDI) mass spectrometry imaging (MSI) technique allowed the visualization of oligosaccharides in plant tissue sections with a lateral resolution of ~40 μm. It was possible to distinguish hexose polymers up to a degree of polymerization (DP) of 11 (Supplementary Figure S2). The sugars, mainly fructans, were identified according to their typical fragmentation pattern, displaying neutral losses of *m/z* 162 (Supplementary Figure S3). 

**Figure 1:**
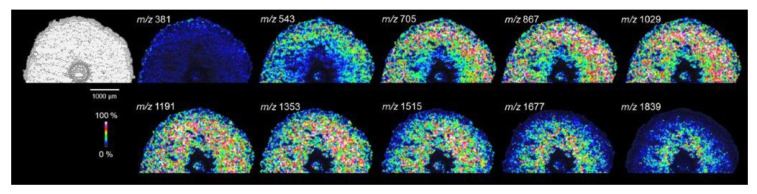
Oligosaccharide distribution patterns obtained by MALDI-MSI. Shown is a light microscopic image of a section together with the extracted ion intensity plots of the molecular ions [M + K]^+^ for sucrose (*m/z* 381) with a degree of polymerization (DP) of 2, as well as for oligosaccharides with DP3 (*m/z* 543), DP4 (*m/z* 705), DP5 (*m/z* 867), DP6 (*m/z* 1029), DP7 (*m/z* 1191), DP8 (*m/z* 1353), DP9 (*m/z* 1515), DP10 (*m/z* 1677), and the polysaccharide with DP11 (*m/z* 1839) for one representative experiment.

An alignment of the MSI data with histological sections indicated a gradient for the root tissue abundance of fructans with different DP (Figure 1). For sucrose (*m/z* 381, DP2) and for oligosaccharides with DP3 (*m/z* 543), a generally higher abundance in the outer tissues and the middle tissues was observed compared to the inner tissues. The molecular ions for fructooligosaccharides with DP4 (*m/z* 705), DP5 (*m/z* 867), DP6 (*m/z* 1029), DP7 (*m/z* 1191), DP8 (*m/z* 1353), and DP9 (*m/z* 1515) appeared uniformly distributed across the root tissues with a slight decrease in abundance towards the inner tissues. Remarkably, for fructooligosaccharides with DP10 (*m/z* 1677) and fructopolysaccharides with DP11 (*m/z* 1839), a higher abundance in the middle and inner tissues was observed. It has to be noted that all molecular ion signals were totally absent for the pericycle and the phloem, while low abundant molecular ion signals for all compounds were observed in the xylem of the inner root tissues.

**Scheme 2:**
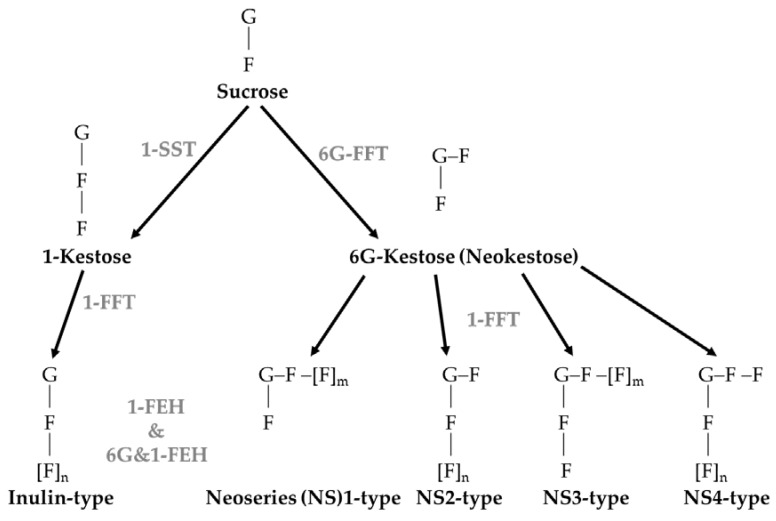
Major fructan types in asparagus. 1-SST (sucrose:sucrose 1-fructosyltransferase) uses sucrose as receptor to form 1-kestose from a sucrose donor with a β (2–1) linkage. 6G-FFT (fructan:fructan 6G-fructosyltransferase) forms 6G-kestose using sucrose as receptor and 1-kestose as donor with a ß (2–6) linkage. 1-FFT (fructan:fructan 1-fructosyltransferase) catalyzes the transfer of fructose from one fructan to another, forming a β (2–1) linkage). Starting from 1-kestose inulin-type fructans are synthesized, while starting from 6G-kestose various inulin neoseries-type fructans are formed in Asparagaceae. Fructan hydrolysis is likely realized by 1-FEH (fructan 1-exohydrolase) and 6G&1-FEH (fructan 6G&1-exohydrolase) as suggested by [9].

It is generally known that asparagus roots accumulate FOS (DP ≤ 10) and fructopolysaccharides (DP > 10) with a DP of up to 22 ([42] and references therein). Asparagus fructans belong to the structural groups of inulin- and inulin neoseries-type fructans [7], whereas according to the position of the attached fructosyl-units, at least four different structures can be assigned to the latter (Scheme 2). By MALDI-MSI technology, such structural particularities cannot be distinguished. In this regard, separation by high pH anion exchange chromatography (HPAEC) coupled to the high sensitivity of pulsed amperometric detection (PAD) is considered the best technique for the analysis of fructans with a DP of up to 80 [5]. Thus, to further investigate the variations in fructan profiles, we have manually isolated three different root tissues, namely outer (O), middle (M) and inner (I) tissues (Supplementary Figure S1). Extracts of soluble carbohydrates from these tissues were then analyzed by HPAEC-PAD.

### 3.2. HPAEC–PAD Analysis Validated Differential Fructan Profiles in the Various Root Tissues

**Figure 2:**
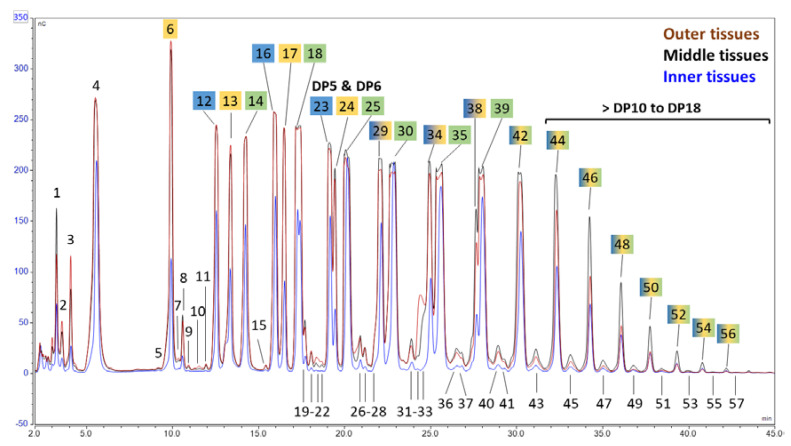
Overlay of tissue oligosaccharide profiles obtained by HPAEC-PAD analysis. Peak numbers refer to annotations listed in Table 1. Peak number color labels refer to inulin-type fructans (yellow), inulin neoseries1-type fructans (blue), and inulin neoseries 2–4-type fructans (green).

**Figure 3:**
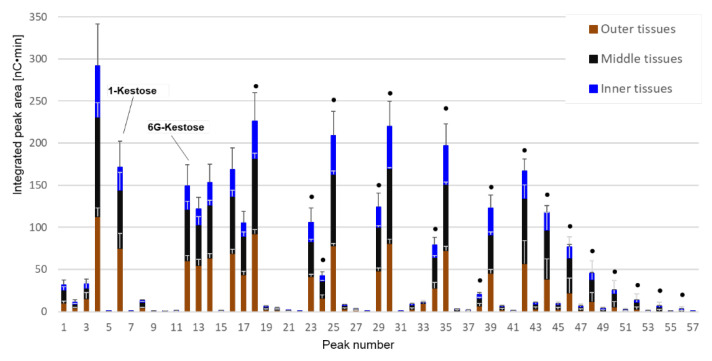
Mean integrated peak areas of oligosaccharide profiles obtained by HPAEC-PAD analysis for the different tissues. Peak numbers refer to annotations listed in Table 1. Mean values and errors were calculated from measurements of the three independent biological replicates. Fructan peaks with increased abundance ratio relative to those of 1-kestose and 6G-kestose for middle and/or inner tissues when compared to outer tissues are highlighted with (•) according to the ratios presented in Table 2. Separate levels for the individual tissues are presented in Figure S4.

The extracts prepared from asparagus root tissues had to be highly diluted for HPAEC-PAD analysis (1:64,000; *w*/*v*, mg/µL) to allow the reliable detection of the high abundant fructooligosaccharides. With this dilution, it was possible to distinguish fructans up to a DP of 17 (Figure 2 and Table 1). Fructopolysaccharides with DP > 17 were barely detectable in root extracts in our measurements. Generally, HPAEC allowed for good separation of the inulin- and inulin neoseries-type fructans up to DP6, whereas we have observed an increased coelution and the decline of the detection limit with increasing DP (Figure 2). Besides the major chromatographic peaks, which could be assigned as the main fructan types known from asparagus (Scheme 2), several smaller peaks were detected. Although these low abundant compounds were hydrolysable by fructanase and mild acid treatment, their structure remains yet unknown. A total of 57 peaks has been annotated (Table 1), and integrated peak areas were extracted for the relative comparison of compound abundance between the different root tissues (Table S1, Figure 3).

The highest amount of carbohydrates was detected for the middle root tissues (total peak area 1342) followed by the outer tissues (1199) and the inner tissues (608), based on the cumulative sum of all 57 integrated peak area values. Similarly, the total peak area for the main fructan peaks was highest for the middle root tissues (1137) followed by the outer tissues (1015) and the inner tissues (520), see Supplementary Table S1. These results are consistent with the highest ion abundance pattern observed for the middle root tissues by our MALDI-MSI analysis (Figure 1). It must be noted that the detected generally low abundance of total carbohydrates and fructans in the inner root tissues is negatively influenced by the high proportion of phloem tissue with almost no accumulation of carbohydrates.

When looking at the levels of the individual compounds, glucose (#1) showed the highest values in the middle tissues, while it was lowest in the inner tissues. Fructose (#2) levels were observed to be almost similar for the outer and middle tissues and were lowest for the inner tissues (Figure 3, Supplementary Table S1). Also, for the two disaccharides, melibiose (#3) and sucrose (#4), similar levels were observed for the outer and middle tissues and lower values were detected for the inner tissues, whereas mean sucrose levels accounted for the highest peak in all root tissues. The trisaccharide raffinose (#5) was only detected in traces in all tissues, while again levels were lowest in the inner root tissues (Figure 3 and Supplementary Figure S4). The values obtained for all other lower abundant peaks, most of them unknown fructans, were smallest in the inner root tissues when compared to the outer and middle tissues (Supplementary Figure S4). Compared to the middle root tissues, values were higher in the outer tissues for peaks #7, #9, #10, #21, and #33, while they were lower for maltose (#8) and all others (Figure 3, Supplementary Figure S4 and Supplementary Table S1).

For the inulin-type fructans 1-kestose (#6, DP3), nystose (#13, DP4) and 1,1,1-kestopentaose (#17, DP5) as well as for 6G-kestose (#12, DP3), similar levels were detected in the outer and middle root tissues, and clearly lower amounts were observed in the inner tissues. Generally, the levels for inulin-type fructans (#6, #13, #17, #24) decreased gradually in all three tissues and were considered to be hardly detectable from DP8 onwards. Notably, for the fructans with DP ≥6 (inulin-type fructan DP6 #24 and mixed peaks #29, #34, #38, #42, #44, #46, #48, #50, #52, #54, and #56) higher values were detected in the middle tissues when compared to the outer tissues. The same trend was observed for the inulin neoseries-type fructans (#25, #30, #35, #39). Based on this observation, we assumed an increased proportion of fructopolysaccharides, mainly inulin neoseries-type fructans, relative to small fructooligosaccharides following a gradient from the outer to the inner root tissues. We could confirm this assumption by calculating the ratio of 1-kestose and 6G-kestose relative to peak areas for fructans with higher degree of polymerization (Table 2). A clear drop in these ratios relative to the results from outer tissues was observed for the middle root tissues from DP6 onwards as well as for the inner root tissues from DP5 onwards for the inulin neoseries-type fructans, highlighted in grey in Table 2 and marked with a point in Figure 3 and Supplementary Figure S4. These results validated the increased intensity of molecular ion signals for fructans with higher DP in the middle and inner root tissues from our MALDI-MSI analysis (Figure 1), which likely relate mainly to inulin neoseries-type fructans.

### 3.3. Proteome Analysis Revealed a Tissue-Specificity in Sucrose Metabolism and Fructan Biosynthesis

The proteome characteristics of asparagus storage root tissues were investigated using a label-free LC-MS approach. The analysis resulted in the identification of 116,003 peptides, which were assigned to 3337 proteins. Principal component analysis revealed a close grouping of technical replicate runs and a clear separation between protein extracts derived from the three root tissues, explaining 46.5% of the observed variation (Figure S5a). The unfiltered data set revealed a higher similarity in protein expression pattern between the middle and the outer region, as compared to the inner region of the root, which was distinctly separated from the other samples (Figure S5b). The data set was filtered according to the parameters described in the Materials and Methods section. Hence, 2400 proteins were subjected to statistical analysis. In pairwise comparisons, 148 proteins were significantly different in their abundance between outer and middle root tissues, 79 proteins between middle and inner root tissues, and 85 proteins differed between inner and outer root tissues, leading to 228 proteins differentially expressed between at least two root tissues (*p* > 0.05, Benjamini–Hochberg corrected for false-discovery rate, Table S2).

**Figure 4:**
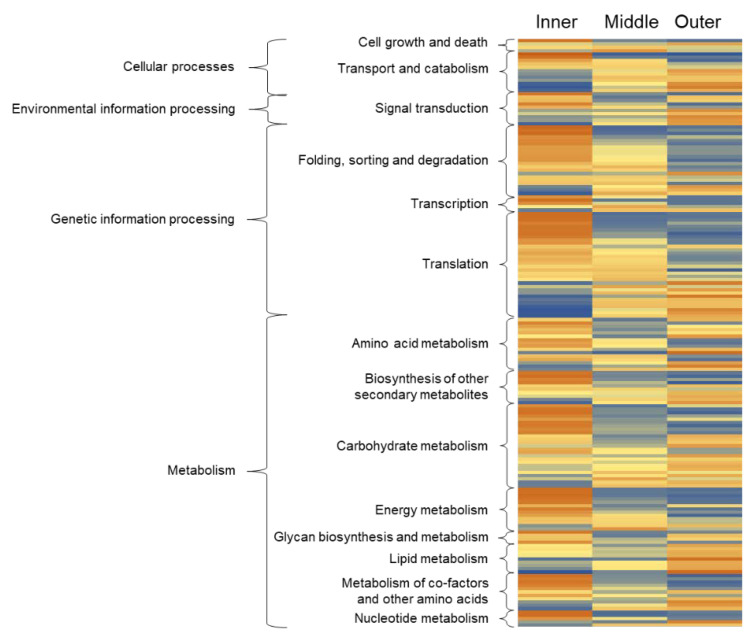
Heatmap depicting differentially abundant proteins that were identified by the label-free proteome analysis. Colors represent the normalized relative protein abundance ranging from zero (dark blue) to 300 (dark orange) for one protein across the different tissues. Functional annotation was performed based on KEGG pathways. Further information related to protein accession numbers, KEGG pathway ID and significance testing is provided in Table S2.

In order to characterize individual protein functions of differentially abundant proteins, KEGG orthology assignments were performed and resulted in the annotation of 77% of all differentially abundant proteins, which was a total of 177 proteins (Figure 4, Table S2). Highest relative abundance in either inner or outer root tissues, when compared with middle root tissues, was detected for almost all assigned proteins of all functional categories. Most proteins involved in ‘folding, sorting and degradation’, ‘amino acid metabolism’, ‘glucan biosynthesis and metabolism’, ‘metabolism of co-factors and other amino acids’, as well as ‘carbohydrate’ and ‘energy metabolism’ were more abundant in the inner root tissue, likely indicating high activity of growth and development-related processes, which rely on the carbon translocation from aboveground plant tissues through the stele and on the substantial generation of energy required for root growth. Many proteins involved in the pathways ‘signal transduction’ and ‘lipid metabolism’ were highly abundant in the outer root tissue, reflecting the function of the epidermis in processing environmental cues and forming of a hydrophobic barrier towards the rhizosphere. Further, several proteins related to ‘transport and catabolism’ were more abundant in the outer tissues, which points to the uptake and release of molecules from and to the rhizosphere, a central root function. Notably, some proteins related to ‘transport and catabolism’ were more abundant in the inner tissues, which likely relates to the transport of water, ions, and sugars from and to the vascular system. Among the functional categories, ‘cell growth and death’, ‘transcription’, ‘translation’ and ‘nucleotide metabolism’ proteins showing the highest relative abundance in either inner or outer root tissues was apparent. Together, these proteome results provide evidence for a high degree of compartmentalization of fundamental metabolic functions between the three asparagus storage root regions investigated.

**Figure 5:**
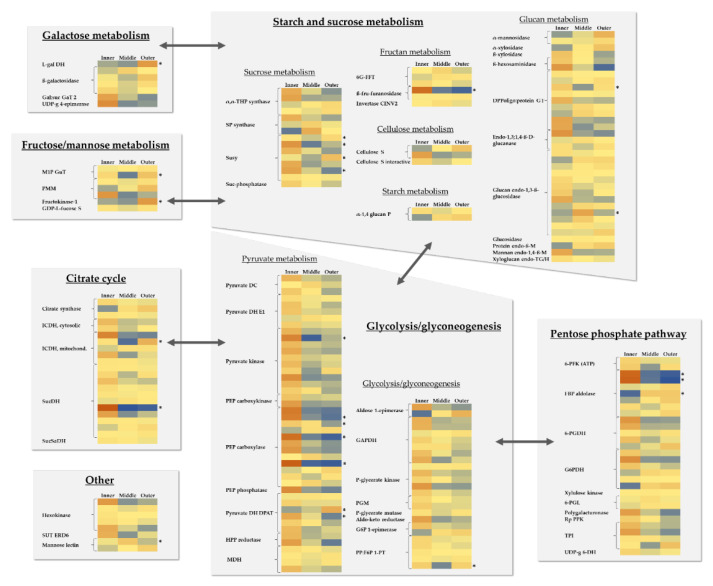
Heatmaps depicting candidate proteins that were identified by the proteome analysis involved in carbohydrate metabolism. Colors represent the normalized relative protein abundance ranging from zero (dark blue) to 300 (dark orange) across the presented data set. Asterisks (*) indicate statistically significant different protein abundances between at least two tissues (*p* < 0.05). Related data from protein analysis are presented in Table S3.

To identify a possible spatial distribution of fructan metabolism, patterns of proteins related to the underlying biosynthetic pathways and related carbohydrate metabolism pathways were further examined. Figure 5 details the abundance pattern of candidate proteins identified by the proteome analysis. Colors represent the normalized relative protein abundance ranging from zero (dark blue) to 300 (dark orange) across the presented data set in order to highlight the strongest differences in abundance.

Six proteins likely involved in ‘fructan metabolism’ were identified. Among them were three proteins annotated as fructan:fructan 6G-fructosyltransferase (6G-FFT), likely representing different isoforms of this enzyme and showing different abundance patterns between the three root tissues (Figure 5, Supplementary Table S3). Two proteins were annotated as ß-fructofuranosidase (ß-fru-furanosidase), which likely exhibit fructan:fructan 1-fructosyltransferase (1-FFT) activity. One of these ß-fructofuranosidases showed very strong and significant differences in abundance between the three root tissues, which was highest in the inner tissues and lowest in the outer tissues. The other ß-fructofuranosidase showed no differences in abundance between the root tissues. Also, an alkaline/neutral invertase CINV2-like protein (invertase CINV2) with yet unknown role in fructan metabolism was identified, showing the highest relative abundance in middle tissues and lowest in outer tissues. The highest absolute amounts, among the known fructan biosynthesis enzymes detected, were observed for two of the 6G-FFT isoforms, while the other 6G-FFT isoform and the two ß-fructofuranosidases showed lower absolute amounts in all three root tissues (Supplementary Figure S6). In addition, we identified a protein annotated as bifunctional 6 (G)-fructosyltransferase/2, 1-fructan: 2, 1-fructan 1-fructosyltransferase (Swissprot ID P92916) in all three root tissues, which probably exhibits 6G-FFT and 1-FFT activity. However, this protein did not pass the threshold of 10% sequence coverage and therefore has not been quantified (please refer to data repository MassIVE https://massive.ucsd.edu/ProteoSAFe/static/massive.jsp?redirect=auth under the dataset ID MSV000085670). Other proteins involved in asparagus fructan biosynthesis (Scheme 2), such as 1-SST, 1-FEH, and 6G&1-FEH, have not been identified in our study.

Several enzymes involved in ‘glucan metabolism’ were observed to exhibit unchanged abundance or lower levels in inner and middle tissues, while others showed similar abundance patterns to the significantly different abundant β-fructofuranosidase, namely much higher in inner tissues and gradually decreased abundance in middle tissues and outer tissues (Figure 5, Table S3). The latter pattern was also observed for one isoform of cellulose synthase (cellulose S) involved in ‘cellulose metabolism’. For ‘starch metabolism’-related proteins, either unchanged abundance among the tissues or highest abundance in outer tissues were observed. For enzymes involved in ‘sucrose metabolism’, several isoforms were detected each, which were observed to show variable abundance patterns between the three root tissues. However, for all enzymes detected, such as alpha, alpha-trehalose-phosphate synthase (α, α-THP synthase), sucrose-phosphate synthase (SP synthase), sucrose synthase (Susy), and sucrose phosphatase (Suc-phosphatase), protein variants with highest abundance in inner root tissues and lowest abundance in outer root tissues were observed. A higher abundance of ‘galactose metabolism’-related enzymes was detected for outer root tissues, with the exception of galactinol:sucrose galactosyltransferase 2 (Gal: suc GaT 2), showing the highest abundance in inner tissues and lowest in outer tissues, and UDP-glucose 4-epimerase (UDP-g 4-epimerase) showing the highest abundance in inner tissues and lowest in middle tissues. Mostly unchanged abundance patterns or lowest abundance in middle root tissues were detected for ‘fructose/mannose metabolism’ related proteins. One phosphomannomutase (PMM) isoform showed the highest levels in inner root tissues, while for fructokinase-1, the highest levels were detected in outer tissues. Notably, most proteins related to ‘glycolysis/glyconeogenesis’ including ‘pyruvate metabolism’ were observed to be most highly abundant in inner root tissues. Adverse abundance patterns, with high levels in outer tissues, were observed for just a few of the isoforms. Similar results were noticed for enzymes of the related ‘pentose phosphate pathway’. Here, strongest differential abundance, with high levels in inner root tissues, was detected for two isoforms of fructose-bisphosphate aldolase (FBP-aldolase), which is required for sucrose synthesis. For all enzymes involved in the ‘citrate cycle’ protein, variants with highest abundance in inner root tissues were identified, emphasizing the strong demand for energy production in this root tissue. A few others showed adverse or unchanged abundance patterns. Similarly, several hexokinase isoforms and the sugar transporter ERD6 (SUT ERD6) were more abundant in the inner root tissues (Figure 5, Table S3).

## 4. Discussion

This study showed that the accumulation of fructans in asparagus storage roots is dependent on the root region and that short chain FOS locate to outer tissues, while long chain FOS and fructopolysaccharides accumulate in the middle and inner tissues of the root cortex. We also demonstrate that specific proteins involved in carbohydrate metabolism and specifically fructan biosynthesis, co-locate with the fructans.

MALDI-MSI has become a valuable alternative to more time-consuming methods, such as laser-capture microdissection or cell sorting, for revealing the tissue-specific spatial localization of compounds. Previous studies proved that asparagus is well suited for MALDI-MSI given the manageable tissue sizes and accumulation of specialized metabolites [43,44]. In addition to that, MALDI-TOF mass spectrometry was applied to classify asparagus storage roots based on their fructan profile [45,46]. The main ion signals recorded from root cross sections in our MALDI-MSI experiments were derived from fructans with DP2 to DP11 (Figure 1, Figure S2), indicating that this method is highly suitable for FOS profile analysis. Although the ion intensities decreased with increasing DP, further MALDI-MSI method improvement should allow the detection of distribution patterns of fructans with DP >11 in the future, given that asparagus roots contain fructans up to a DP of 22 ([42] and references therein, [46]). In our study, the combination with HPAEC-PAD analysis enabled the detection and quantification of fructans up to a DP of 17, as well as the differentiation of the various fructan types (Figure 2). Therefore, we could assign the observed increased proportion of fructopolysaccharides, relative to small FOS, following a gradient from the outer to the inner root tissues, mainly to inulin neoseries-type fructans (Figure 3). This highlights the demand of combining various analytical measures to elucidate specific abundance patterns of different fructan types [5].

The spatiotemporal variation in the accumulation of different fructan types was also reported from other species and organs, such as barley grain [17,35], and points towards versatile roles of the different fructan types. Thus, we hypothesize that such particularities of fructan biosynthesis may have evolved differently in several plant species. The accumulation of inulin neoseries-type fructans with higher DPs near the vasculature of asparagus storage roots, found in our study, could indicate a protective mechanism to maintain long-distance transport. It has been shown that especially long-chain fructans accumulate upon cold treatment in ryegrass crowns, which is the basal zone of the shoot and contains over-wintering tissues [47]. This indicates a function in freezing tolerance, which is essential for perennial plant species. In asparagus, root fructan content decreased when plants were transferred from cold storage to greenhouse conditions, which also points towards a protective role of fructans during cold stress in tissues that need to remain vital during winter season [9]. Histochemical and scanning electron microscopy (SEM) analyses of *Campuloclinium chlorolepis* tuberous roots, provided information on the spatial distribution of fructans at cellular and subcellular levels [25]. The presence of fructans, mainly inulin-type, was assessed inside and outside the cells of all tissues of tuberous roots except for the epidermis. SEM allowed for the detection of globular bodies consistent with typical inulin spherocrystals observed under polarized light. These globular bodies varied in size according to location, being smaller in the cortical tissue and larger in the central cylinder. From the results, interaction of fructans with the cell membrane and a possible role in membrane stabilization in the central cylinder has been proposed. Similar observations were reported for *Taraxacum officinale,* indicating that fructans (with DP3 to DP50) are preferentially stored in phloem parenchyma cells in the vicinity of the secondary sieve tube elements, whereas inulin sperocrystals occasionally appeared also in xylem vessels [24]. In contrast, increased levels of short-chain inulin-type fructans in transfer tissues of barley grain have been proposed to protect the membranes during active sucrose and nutrient transport [17]. In this regard, the accumulation of inulin-type fructans with DP <6 in outer and middle root tissues may protect those cells during water and nutrient transport from the soil towards the central cylinder, while the increased levels of inulin neoseries-type fructans with higher DP in the inner tissues may represent an efficient way of carbon partitioning. Accumulation of inulin neoseries-type fructans in the inner root tissues of asparagus may be related to favorable structural characteristics when compared to inulins and to the function of this tissue as a storage of carbohydrates for growth of the spears and the asparagus stand. Some reports showed that fructan branching architecture is critical to physicochemical properties. Comparing inulin and sinistrin (a neoseries-type fructan), sinistrin showed higher alkali-resistance and better water solubility [48]. In experiments with agave extracts, containing highly branched and high DP agavins, parameters like density, molar volume, pH, conductivity, particle size, and viscosity were strongly influenced by the concentration, suggesting that fructans can probably form aggregates at higher concentrations [49]. Analysis of polydispersity of two inulin-type fructans with the same number of branches but potentially different branching architectures (short versus long chain branched) revealed similar compact globular shapes, indicating strong intramolecular interactions between more distant residues of the same molecule [50]. A more compact shape of neoseries-type fructans would allow higher concentrations to be stored in the vacuole. Better water solubility and pH-stability of neoseries-type fructans would be advantageous for quick enzymatic mobilization during growth (e.g., fructans to be degraded to fructose and glucose), such as proposed for neoseries-type fructans accumulating in the aleurone of barley grain [35]. Proving these hypotheses will require the comparative evaluation of physicochemical properties of neoseries- and inulin-type fructans in the future.

With the aim to understand the precise contribution of the root cell types to fructan accumulation, a label-free proteome analysis was performed from three regions from root transversal sections. By cell type separation, we reduced the complexity of the total root proteome, allowing the reliable identification of 3337 proteins. The analysis revealed a higher similarity between the outer (epidermis, exodermis and cortex) and middle region (cortex) as compared to the inner region (cortex, endodermis, stele). The protein abundance patterns reflected the differential functions of the root tissues (Figure 4), e.g., the processing of environmental cues and forming of a hydrophobic barrier towards the rhizosphere, as well the uptake and release of molecules from and to the rhizosphere in the outer and middle root tissues, and the high activity of transport from and to the vascular system and processes related to growth and development in the inner tissue. Previously, molecular functions of distinct root cell types have been studied on the transcriptome level in *Arabidopsis thaliana* using cell sorting in detail [51,52,53,54,55]. However, comparable proteome studies have been missing until now. Pioneering work on proteomics of tomato and maize root cell types excised via laser capture microdissection demonstrated the potential to describe their molecular functions [32,56,57]. Our study can further aid to unravel biochemical and molecular processes in the root by providing a resource of root cell type-specific proteomic data (MassIVE dataset ID MSV000085670). We found that many proteins related to carbohydrate metabolism, particularly related to balancing sucrose concentrations, were highly abundant in the inner root tissue (Figure 5). Generally, sucrose levels accounted for the highest peak in all three root tissues, while highest values were observed in middle and outer root tissues. In plants, sucrose is transported from source to sink organs over long distances in solution in the phloem sap via the network of sieve element cells [58]. As sucrose must pass several membrane barriers on its way, several sucrose transporters were shown to be involved in this process [59]. The high concentration of sucrose in the sieve elements increases turgor pressure, allowing sucrose transport to the sink organs according to the Mass-Flow model [60]. Unloading of sucrose from the phloem can occur through symplastic or apoplastic pathways depending on tissue types or development stages, but always where the turgor pressure drops. Apoplastic sucrose unloading involves sucrose transporters in sink organs or its conversion to hexoses by a cell-wall invertase (please refer to [59] and references therein). Therefore, central vessel cells in asparagus roots, including sieve elements, will force sucrose to be released from the inner root tissues towards the outer root tissues. It is very likely that for this purpose, the sucrose concentration, and thus turgor pressure, must be kept low in the tissues adjacent to the central vessel cells. This fits well to our observation that for all ‘sucrose metabolism’-related enzymes isoforms with highest abundance in inner root tissues and lowest abundance in outer root tissues were detected. Accordingly, for almost all other ‘carbohydrate metabolism’-related enzymes, highest abundance levels in inner tissues were detected in our study. Among the six proteins involved in ‘fructan metabolism’, two proteins showed highest expression in the inner root tissue. One was a 6G-FFT (Swissprot ID Q5FC15), involved in the formation of neoseries-type fructans and described in asparagus previously [10]. The other one was assigned as ß-fructofuranosidase (NCBI ID gi: 1150742863), derived and annotated from a genomic sequence from asparagus, which has not yet been functionally characterized and revealed a highly significant differential expression. The enzyme product of this gene likely exhibits fructan:fructan 1-fructosyltransferase (1-FFT) activity, mediating the transfer of fructose units from one fructan to another and thus leading to diminishment of sucrose and to the enhanced formation of higher DP inulin neoseries-type fructans in the inner root tissues. Other fructan biosynthesis genes described from Asparagales, such as 1-SST and 1-FEH [61], as well as 6G&1-FEH from asparagus, have not been identified in our study, which could be due to differences in plant age [9]. Our results demonstrate that cell type-specific proteome analyses are particularly useful for studying the molecular mechanisms of processes, which have varied effects on different layers of root cells.

## Figures and Tables

**Table 1:** Oligosaccharide annotation information. The 57 metabolites are listed with their retention time (RT), the determined degree of polymerization (DP), the molecular family and fructan-type and the main molecular ion [M + K]^+^ determined by mass spectrometry (MS).

Peak #	RT [min]	Compound	DP	Molecular Family/Fructan-Type	[M + K]^+^
1	3.24	Glucose	1	Monosaccharide	
2	3.56	Fructose	1	Monosaccharide	
3	4.08	Melibiose	2	Disaccharide	381
4	5.49	Sucrose	2	Disaccharide	381
5	9.16	Raffinose	3	Raffinose-type oligosaccharides	543
6	9.84	1-Kestose	3	Inulin-type fructan	543
7	10.35	Unknown			
8	10.58	Maltose	2	Maltose-type oligosaccharides	
9	10.93	Unknown			
10	11.59	Unknown			
11	11.94	Unknown			
12	12.51	6G-Kestose (NS1-DP3)	3	Neoseries (NS)1-type fructan	543
13	13.35	Nystose (1,1-Kestotetraose, KT) (Inulin-DP4)	4	Inulin-type fructan	705
14	14.20	1&6G-KT (NS2-DP4)	4	NS2-type fructan	705
15	15.40	Unknown			
16	15.83	1, 6G-KT (NS1-DP4)	4	NS1-type fructan	705
17	16.45	1,1,1-Kestopentaose (KP) (Inulin-DP5)	5	Inulin-type fructan	867
18	17.13	1,1&6G-KP (NS2-DP5), 1&1, 6G-KP (NS3-DP5)	5	NS2&NS3-type fructan	867
19	17.69	Unknown			
20	18.04	Unknown			
21	18.33	Unknown			
22	18.66	Unknown			
23	19.03	1,1, 6G-KP (NS1-DP5)	5	NS1-type fructan	867
24	19.43	1,1,1,1-Kestohexaose (KH) (Inulin-DP6)	6	Inulin-type fructan	1029
25	20.04	1,1,1&6G-KH (NS2-DP6), 1&1,1, 6G-KH (NS3-DP6), 1,1&1, 6G-KH (NS4-DP6)	6	NS2-4-type fructan	1029
26	20.89	Unknown			
27	21.17	Unknown			
28	21.71	Unknown			
29	22.11	1,1,1, 6G-KP (NS1-DP6) and Inulin-DP7	6&7	Inulin & NS1-type fructan	1029&1191
30	22.92	1,1,1,1&6G-KH (NS2-DP7), 1&1,1,1, 6G-KH (NS3-DP7), 1,1,1&1, 6G-KH (NS4-DP7)	7	NS2-4-type fructan	1191
31	23.42	Unknown			
32	23.88	Unknown			
33	24.23	Unknown			
34	24.95	NS1-DP7 and Inulin-DP8	7&8	Inulin & NS1-type fructan	1191&1353
35	25.64	NS2-DP8, NS3-DP8, NS4-DP8	8	NS2-4-type fructan	1353
36	26.50	Unknown			
37	26.84	Unknown			
38	27.65	NS1-DP8 and Inulin-DP9	8&9	Inulin & NS1-type fructan	1353&1515
39	28.04	NS2-DP9, NS3-DP9, NS4-DP9	9	NS2-4-type fructan	1515
40	28.93	Unknown			
41	29.32	Unknown			
42	30.22	NS1-DP9, Inulin-DP10, NS2-DP10, NS3-DP10, NS4-DP10	9&10	Inulin & NS1-NS4-type fructan	1515&1677
43	31.13	Unknown			
44	32.33	NS1-DP10, Inulin-DP11, NS2-DP11, NS3-DP11, NS4-DP11	10&11	Inulin & NS1-NS4-type fructan	1677&1839
45	33.13	Unknown			
46	34.27	NS1-DP11, Inulin-DP12, NS2-DP12, NS3-DP12, NS4-DP12	11&12	Inulin & NS1-NS4-type fructan	
47	35.02	Unknown			
48	36.08	NS1-DP12, Inulin-DP13, NS2-DP13, NS3-DP13, NS4-DP13	12&13	Inulin & NS1-NS4-type fructan	
49	36.78	Unknown			
50	37.77	NS1-DP13, Inulin-DP14, NS2-DP14, NS3-DP14, NS4-DP14	13&14	Inulin & NS1-NS4-type fructan	
51	38.42	Unknown			
52	39.33	NS1-DP14, Inulin-DP15, NS2-DP15, NS3-DP15, NS4-DP15	14&15	Inulin & NS1-NS4-type fructan	
53	39.93	Unknown			
54	40.81	NS1-DP15, Inulin-DP16, NS2-DP16, NS3-DP16, NS4-DP16	15&16	Inulin & NS1-NS4-type fructan	
55	41.42	Unknown			
56	42.20	NS1-DP16, Inulin-DP17, NS2-DP17, NS3-DP17, NS4-DP17	16&17	Inulin & NS1-NS4-type fructan	
57	42.81	Unknown			

# refers to “number”.

**Table 2:** Ratio of 1-kestose and 6G-kestose relative to peaks for fructans with higher degree of polymerization. Peak numbers refer to annotations in Table 1. Ratios were calculated from mean integrated peak area shown in Figure 3 and Supplementary Table S1. Values for middle and inner tissues, which had decreased by at least 20% (light gray) or at least 50% (dark gray) relative to outer tissues, are highlighted.

	Peak	13	14	16	17	18	23	24	25	29	30	34	35	38	39	42	44	46	48	50	52	54	56
**Outer tissues**	**1-Kestose**	1.4	1.2	1.1	1.7	0.8	1.8	4.7	1.0	1.6	0.9	2.7	1.0	11.0	1.7	1.3	1.9	3.4	6.4	13.0	28.8	61.7	132.0
	**6G-Kestose**	1.1	1.0	0.9	1.4	0.7	1.5	3.8	0.8	1.3	0.7	2.2	0.8	8.8	1.3	1.1	1.6	2.7	5.2	10.5	23.2	49.6	106.2
**Middle tissues**	**1-Kestose**	1.4	1.1	1.0	1.5	0.8	1.7	3.4	0.8	1.3	0.8	1.9	0.9	7.6	1.5	0.9	1.2	1.7	2.6	4.4	8.0	15.2	29.3
	**6G-Kestose**	1.3	1.0	0.9	1.3	0.7	1.5	3.0	0.7	1.2	0.7	1.7	0.8	6.7	1.3	0.8	1.0	1.5	2.3	3.9	7.1	13.4	25.9
**Inner Tissues**	**1-Kestose**	1.5	1.0	0.9	1.8	0.6	1.2	4.5	0.6	1.1	0.5	1.9	0.6	6.3	0.9	0.8	1.3	2.1	3.7	6.7	13.0	25.2	48.7
	**6G-Kestose**	1.5	1.0	0.9	1.8	0.6	1.2	4.5	0.6	1.2	0.5	1.9	0.6	6.3	0.9	0.8	1.3	2.1	2.8	7.0	13.3	25.6	49.5

## Supplementary Materials

The following are available online at https://www.mdpi.com/2073-4409/9/9/1943/s1, Figure S1: Sample preparation from asparagus roots, Figure S2: Average mass spectrum from the matrix-assisted laser desorption ionization (MALDI) mass spectrometry (MS) analysis, Figure S3: Mass fragmentation analysis of the detected main molecular ions, Figure S4: Individual view of mean integrated peak areas of oligosaccharides obtained by HPAEC-PAD analysis for the different root tissues, Figure S5: Assessment of technical and biological variation in protein expression profiles of the three root tissues, Figure S6: Abundances values of identified proteins known to be involved in fructan metabolism, Table S1: Integrated peak areas of oligosaccharide profiles obtained by HPAEC-PAD analysis for the different root tissues. Table S2: Identification and functional classification of proteins that were significantly differentially abundant in the three analysed regions of asparagus roots. Provided are lists of all identified proteins (spreadsheet “identified”), statistically significant differentially abundant proteins (spreadsheet “differentially”) and functional classifications (spreadsheet “KEGG”). Table S3: Results related to carbohydrate metabolism from protein profiling analysis of the different root tissues. The proteomics data are available via MassIVE (https://massive.ucsd.edu/ProteoSAFe/static/massive.jsp?redirect=auth) with identifier MSV000085670 under project title ‘Fructooligosaccharides are differentially distributed in root tissues of asparagus’.
