# Supplementary material for: Fructans Are Differentially Distributed in Root Tissues of Asparagus
Source: Cells. 2020 Aug 22;9(9):1943. doi: 10.3390/cells9091943 (PMC7565981; doi:10.3390/cells9091943)
Supplement: Supplementary file 1 [file cells-09-01943-s001.zip › Supplementary Figures.pdf]

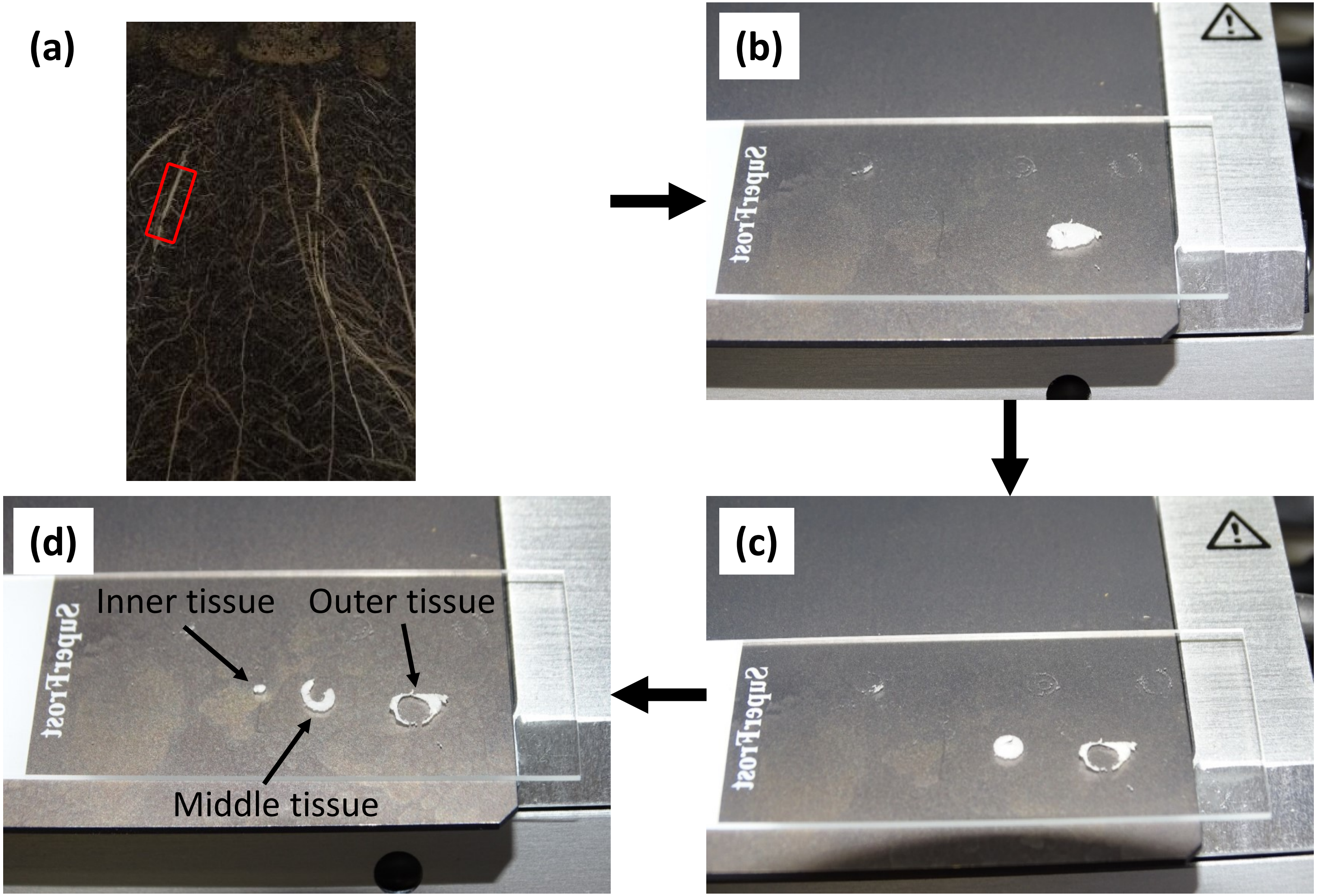

Supplementary Figure S1. Sample preparation from Asparagus roots. (a) Samples were taken from storage roots of two-month-old plants, as indicated by the red rectangle. (b) Manual sections of 150 µm thickness were prepared with a microtome. (c) With a cork borer middle and inner tissues were separated from the section. (d) Then the inner tissues were separated from the middle tissues by a smaller cork borer. In this way outer tissues (O), middle tissues (M) and inner tissues (I) were prepared from three biological replicates in three technical replicates.

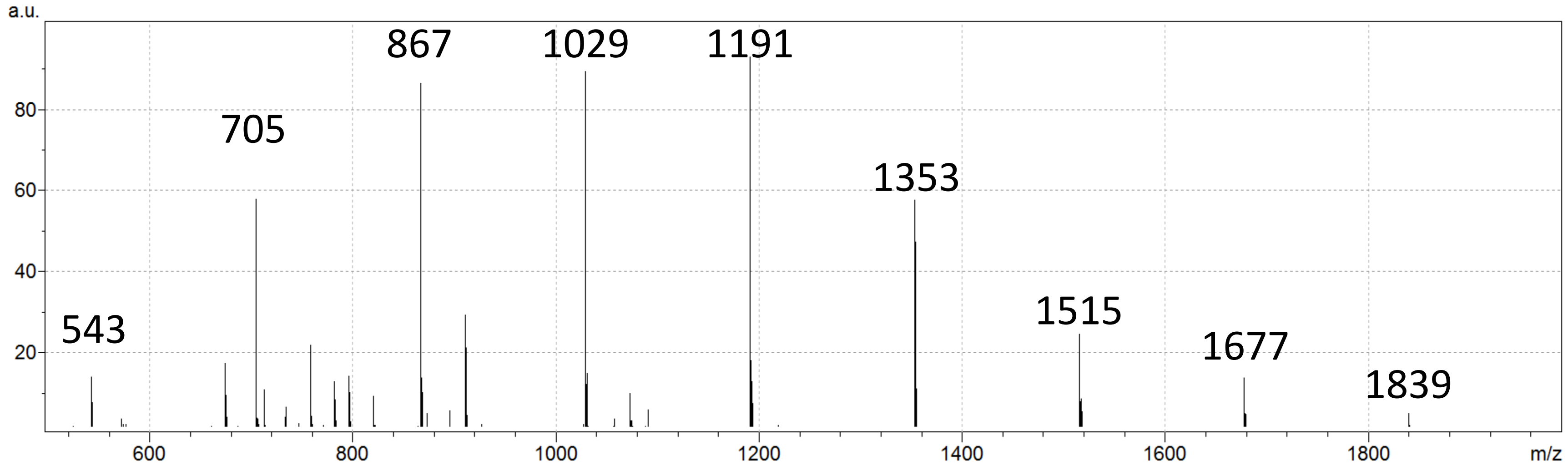

Supplementary Figure S2. Average mass spectrum from the matrix-assisted laser desorption ionisation (MALDI) mass spectrometry (MS) analysis. Molecular ions $[M+K]^+$ for sucrose (*m/z* 381) with a degree of polymerisation (DP) of 2, as well as for oligosaccharides with DP3 (*m/z* 543), DP4 (*m/z* 705), DP5 (*m/z* 867), DP6 (*m/z* 1029), DP7 (*m/z* 1191), DP8 (*m/z* 1353), DP9 (*m/z* 1515), DP10 (*m/z* 1677), and DP11 (*m/z* 1839) are indicated. Corresponding ion intensity plots of the oligosaccharide-related mass signals are presented in Figure 2.

Detected molecular ions [*m/z*]	Detected fragment ions [*m/z*]	DP
543	381	3
705	381, 543	4
867	381, 543, 705	5
1029	381, 543, 705, 867	6
1191	381, 543, 705, 867, 1029	7
1353	381, 543, 705, 867, 1029, 1191	8
1515	381, 543, 705, 867, 1029, 1191, 1353	9
1677	381, 543, 705, 867, 1029, 1191, 1353, 1515	10
1839	381, 543, 705, 867, 1029, 1191, 1353, 1515, 1677	11

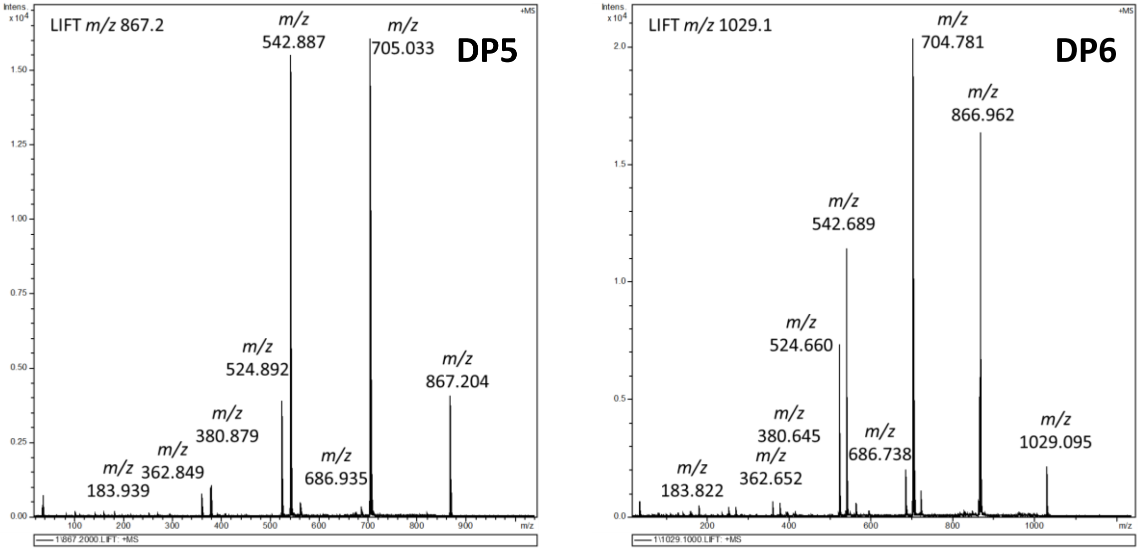

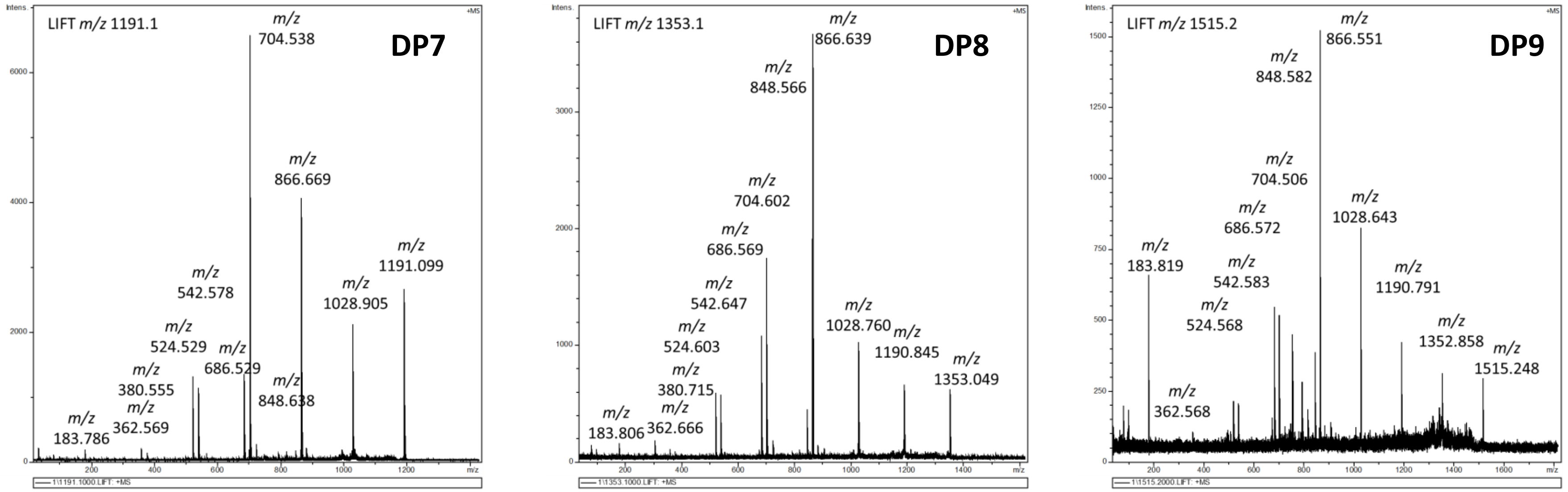

Supplementary Figure S3. Mass fragmentation analysis of the detected main molecular ions. Molecular ions $[M+K]^+$ for oligosaccharides with degree of polymerisation (DP) 3 (*m/z* 543), DP4 (*m/z* 705), DP5 (*m/z* 867), DP6 (*m/z* 1029), DP7 (*m/z* 1191), DP8 (*m/z* 1353), DP9 (*m/z* 1515), DP10 (*m/z* 1677), and DP11 (*m/z* 1839) have all been assigned as fructans due to the release of fructose units upon fragmentation. Corresponding fragment ion mass spectra are shown for DP5 to DP9.

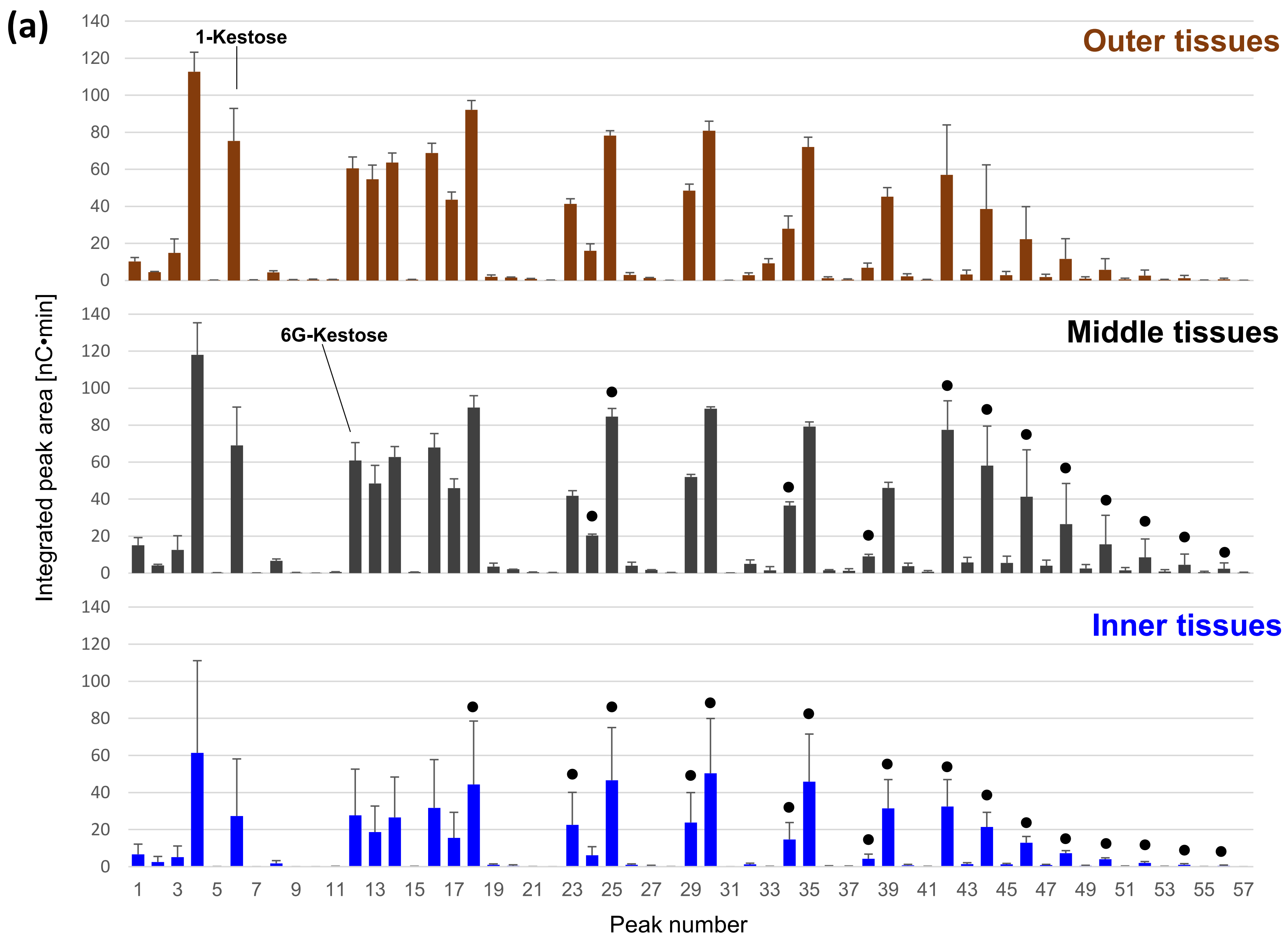

(a)
Outer tissues
1-Kestose
Middle tissues
6G-Kestose
Inner tissues
Integrated peak area [nC•min]
Peak number
0
20
40
60
80
100
120
140
1
3
5
7
9
11
13
15
17
19
21
23
25
27
29
31
33
35
37
39
41
43
45
47
49
51
53
55
57

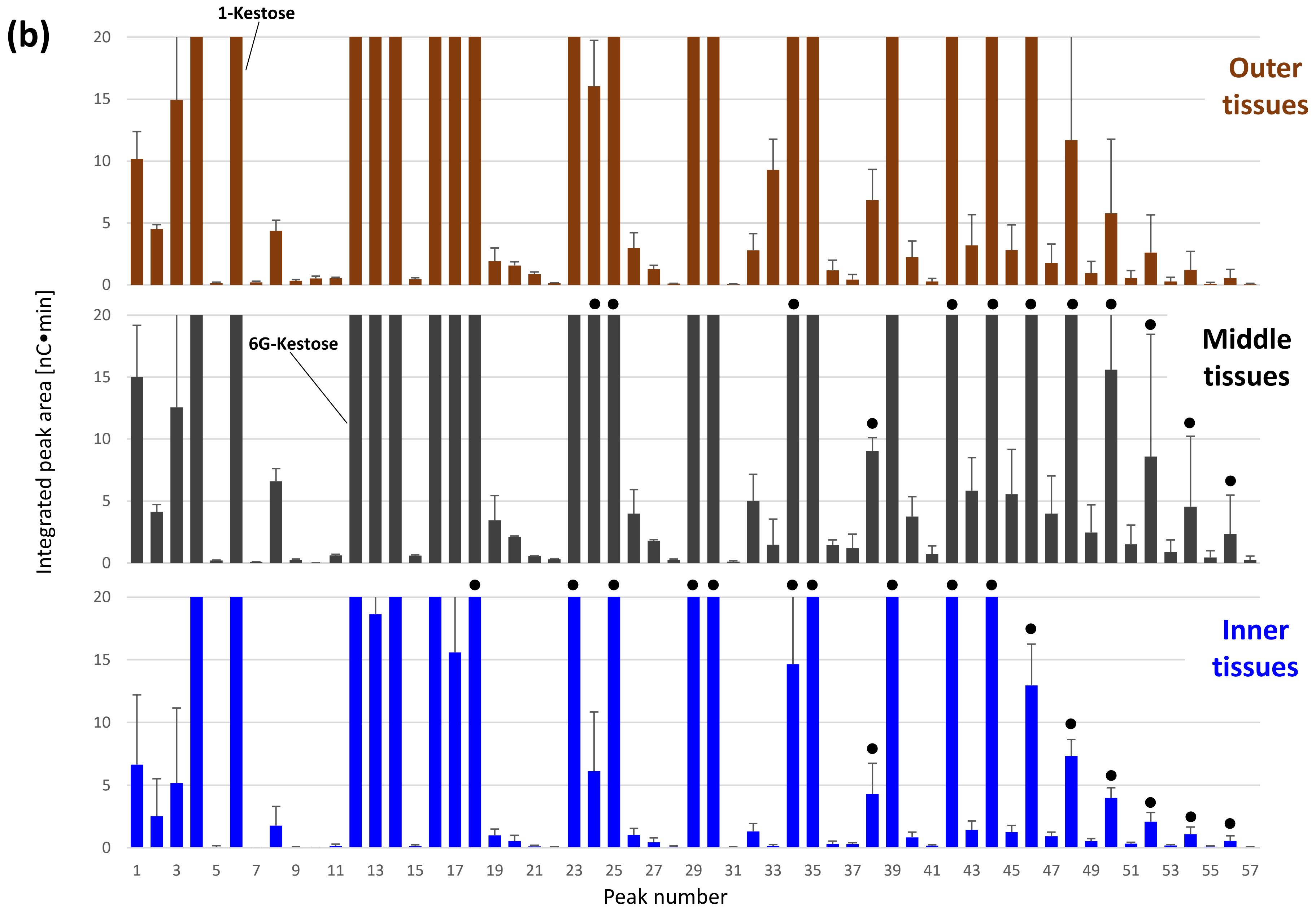

Supplementary Figure S4. Individual view of mean integrated peak areas of oligosaccharide profiles obtained by HPAEC-PAD analysis for the different tissues. (a) shows a total overview and (b) the enlarged view to highlight the lower abundant compounds. Peak numbers refer to annotations listed in Table 1. Fructan peaks for middle and inner tissues with increased abundance ratio relative to those of 1-kestose and 6G-kestose when compared to outer tissues are highlighted with (•) according to the ratios presented in Table 2.

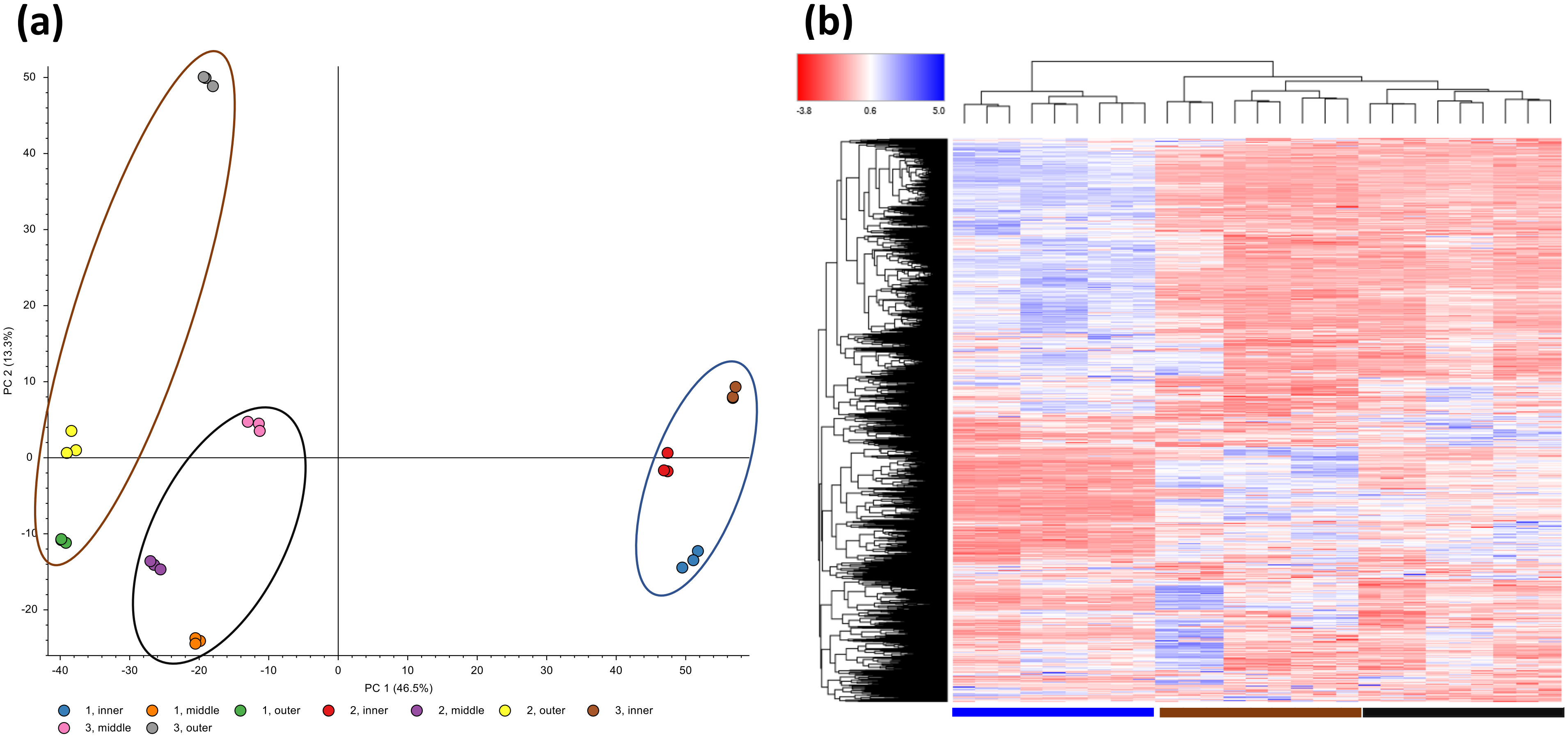

Supplementary Figure S5. Assessment of technical and biological variation in protein expression profiles of the three root tissues (three independent experiments, measured in triplicate LC runs) from asparagus roots using principal component analysis (a). Hierarchical clustering of expression patterns of all identified proteins based on Euclidean distance and complete linkage (b). Each column represents one LC run prepared from samples of inner (blue), middle (black) and outer (brown) tissue regions, in three biological replicates. Rows display the abundance of the proteins using color coding based on a relative scale (–3.8 to +5.0).

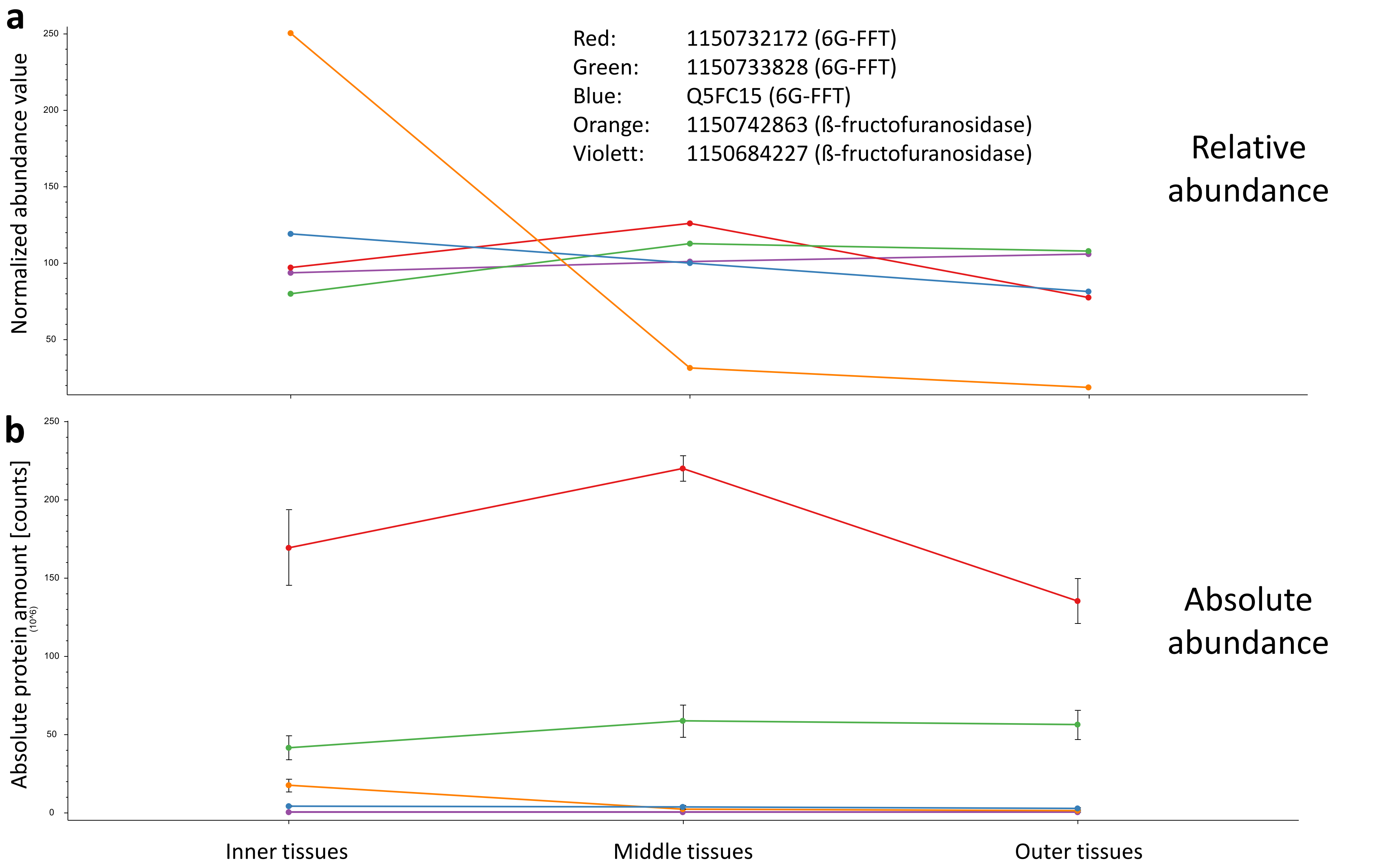

Supplementary Figure S6. Abundance values of identified proteins known to be involved in fructan metabolism. (a) Relative abundance values normalized to the total peptide amount and scaled to the average abundance of 300 and (b) absolute abundance values normalized to the total peptide amount only. The relative protein abundance is calculated for one particular protein as mean of the sum of the peptide group abundances associated with that protein per tissue and then normalized from zero to 300 between the three tissues. The absolute protein abundance is calculated as the sum of the peptide group abundances associated with that protein and then normalized to the total peptide amount of that sample (biological replicate). Error bars represent the variation among the biological replicates.
